# Dopamine as a teaching signal: understanding its role in shaping individual behavior

**DOI:** 10.1038/s41392-025-02406-5

**Published:** 2025-09-05

**Authors:** Seung Chan Kim, Sehwan Kim, Sang Ryong Kim

**Affiliations:** 1 Brain Science and Engineering Institute, Kyungpook National University, Daegu, Korea; 2 School of Life Science and Biotechnology, BK21 FOUR KNU Creative BioResearch Group, Kyungpook National University, Daegu, Korea

**Keywords:** Molecular neuroscience, Assay systems, Predictive markers, Predictive markers

In a recent publication in *Cell*, Liebana and colleagues demonstrate that dopamine acts as a circuit-specific teaching signal, shaping individual learning trajectories over time. These reward prediction error (RPE) signals refine behavior by selectively reinforcing neural pathways, revealing a key mechanism through which dopamine guides personalized learning strategies beyond classical reward-based models.^1^

Dopamine is traditionally known for encoding reward prediction errors, a core component in short-term learning^2^ (e.g., behavioral fine-tuning). However, this classical framework does not fully explain the complexity and individuality of long-term skill acquisition. Real-world learning tasks, such as playing a musical instrument or mastering a sport, often unfold over extended timescales and involve distinct, strategy-dependent phases that vary across individuals. Meanwhile, traditional reinforcement learning (RL) models, which rely on fixed state representations and global value updates, fail to capture such strategic diversity. Similarly, most neuroscience studies have emphasized expert performance rather than the developmental processes that lead to expertise, leaving a critical gap in our understanding of long-term learning trajectories.

Liebana et al. outlined a dynamic role for dopamine, particularly within the dorsolateral striatum (DLS),^3^ in shaping individualized long-term learning.^1^ Using longitudinal behavioral tracking and real-time dopamine measurements, the authors explained how dopaminergic signals evolve throughout learning. Initially, dopamine signals reflect reward outcomes, consistent with classical RPEs. However, as training progresses, the signals gradually shift toward task-relevant stimuli in a manner dependent on the animals’ early behavioral biases and subsequent diverse solution strategies. These biases lead to strategy-specific dopamine dynamics: asymmetric activity in one-sided learners and symmetrical patterns in balanced learners. Importantly, dopamine encoded stimulus-choice associations contingent on the internal learning state of each animal, rather than the learned value of the stimulus as in traditional RL models.

The learning paradigm involved training mice on a visual decision-making task over several weeks. In each trial, the mice were required to turn a wheel to the clockwise or anticlockwise, depending on the location of a visual grating stimulus. The task structure was held constant throughout training, allowing researchers to isolate internally driven learning processes. Behavioral analyses revealed substantial strategic variability: while some mice developed balanced stimulus–response mappings, others adopted highly lateralized strategies, consistently associating one stimulus side with reward and not the other. Notably, early behavioral biases reliably predicted the eventual psychometric performance curve for each animal across the learning phases. Despite following different trajectories, most animals converged on comparable final performance levels, suggesting that diverse structured and optimized strategies can emerge through consistent experience.

To assess the causal role of DLS dopamine in shaping these strategies, the researchers performed optogenetic manipulations during the training period. Inhibition of dopamine release impaired the formation of steep psychometric slopes and stimulus-guided decision-making, despite intact motor execution and sustained task engagement. In a separate experiment, stimulating dopamine in expert one-sided animals following incorrect choices modulated subsequent behavior, only when the animal had used the corresponding stimulus to guide its choice. Conversely, stimulation had no effect when the stimulus was irrelevant to the decision. These findings indicate that dopamine in the DLS serves as a stimulus-contingent teaching signal, and that is engaged selectively when a stimulus is utilized for decisions. In contrast to classical RPEs, which update value representations independent of behavioral context, this dopaminergic signal operates in a more targeted, stimulus- and strategy-specific manner.

To computationally model these findings, they developed a biologically inspired deep RL framework, the Tutor–Executor model. This architecture comprises parallel pathways for sensory and contextual information, incorporating three forms of RPEs. Crucially, the model implements partial, input-specific RPEs, updating only the connections associated with either sensory or contextual inputs, recapitulating the DLS dopamine selectivity observed experimentally. The Tutor–Executor model successfully reproduced key behavioral features observed in mice, such as asymmetric slope development, early behavioral biases, asymmetric slope development and the diverse yet systematic learning trajectories across mice. Dopamine-like signals derived from the learning gradients in the model closely matched the dynamics of recorded DLS dopamine release. Further analysis of the model revealed that transitions between strategies were governed by unstable saddle points in the weight space. These saddle points created temporary learning plateaus, which helped explain why some animals stalled at intermediate learning stages. The model also captured a gradual shift from cortical to subcortical control over time, aligning with biological transitions from goal-directed to habitual behavior.

This study builds on the classical view that dopamine merely encodes RPEs and proposes a broader, more dynamic role in guiding individualized long-term learning. By tracking mice over several weeks during a visual decision-making task, the authors uncovered diverse and structured learning strategies determined by early biases. Dopamine release in the DLS evolved alongside these behavioral shifts, encoding not just reward-based but also strategy-dependent stimulus-choice associations that anticipated future transitions. These signals were causally required for effective strategy formation, operating selectively when animals engaged with decision-relevant information.

This research formulates new directions in neuroscience by highlighting dopamine as a stimulus-contingent teaching signal rather than a global reinforcer. Nonetheless, other brain regions rich in dopaminergic inputs remain to be explored. The RPE model employed in this study can also be extended to other neurotransmission systems involved in associative and reinforcement learning, such as glutamatergic and GABAergic (gamma-aminobutyric acid) circuits, offering broader insights into large-scale learning mechanisms.^4^ Moreover, the Tutor–Executor computational model provides a biologically plausible framework for understanding how partial, input-specific RPEs shape strategy development and adaptive behavior.

**Fig. 1:**
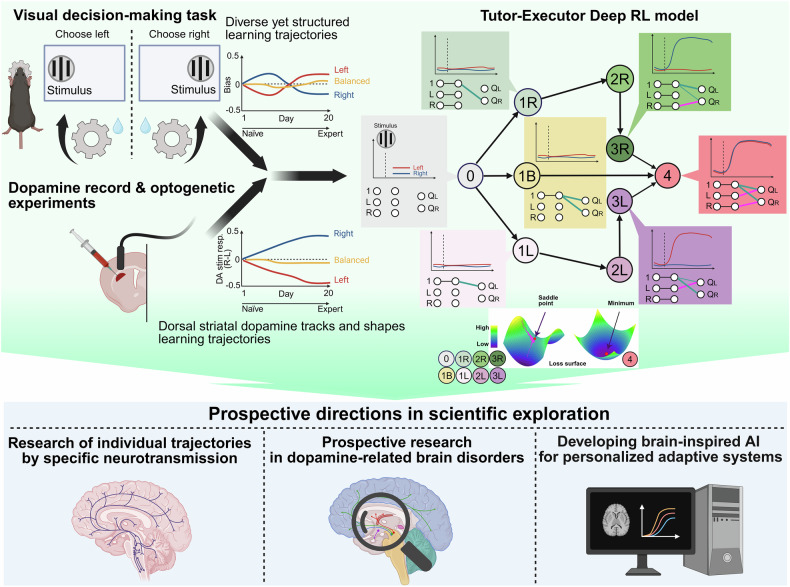
A schematic overview of the role of dopamine in individualized long-term learning (Created and modified using BioRender.com license no: *SV28KABRLC*). Recent work by Liebana et al. demonstrates that, unlike traditional reinforcement learning (RL) models based on value updating with fixed state representations, mice trained on a visual decision-making task developed diverse and structured behavioral strategies, ranging from balanced to strongly biased. Dopamine release in the dorsolateral striatum (DLS) evolved in parallel with these behavioral patterns, encoding stimulus-choice associations in a strategy-dependent manner. Optogenetic manipulations confirmed the causal role of DLS dopamine in guiding strategy formation, specifically when animals were actively engaged with task-relevant stimuli. To model these observations, the authors proposed a biologically inspired Tutor–Executor neural network model that incorporates partial, input-specific reward prediction errors (RPEs), successfully capturing both behavioral and neural dynamics. These findings position dopamine as a stimulus-contingent teaching signal, providing a foundation for future studies to explore similar mechanisms in other brain regions, neuromodulatory systems, and learning contexts. Moreover, this work advances our understanding of neurological and psychiatric disorders associated with dopaminergic signaling and reward-driven behavior, offering both predictive and therapeutic insights. Finally, the principles uncovered here may also inform the development of personalized and adaptive decision-making technologies in artificial intelligence (AI)

These findings may broaden the scope of application beyond fundamental neuroscience. The model can provide insights into disorders and diseases relevant to reward-driven behavioral motivations, such as addiction, depression, or schizophrenia, as well as those involving dopaminergic signalings, including Parkinson’s disease, attention deficit/hyperactivity disorder, or obsessive-compulsive disorder. Moreover, the model enables disease prediction and therapeutic strategies based on individual behavioral and dopaminergic trajectories. The principles can also inform deep RL models in artificial intelligence (AI), enhancing decision-making and error correction through feedback, mirroring the brain’s mechanisms.^5^ Such AI-based models could deliver personalized experiences in education, training, and daily decision-making, even suggesting music to match the mood of the individual. Together, these findings on DLS dopaminergic signaling and the “Tutor–Executor” model will enhance our understanding of how we perceive and adapt to the world, highlighting how insights into the brain’s learning mechanisms can teach us the optimization of learning strategies in animals, humans and AI models (Fig. 1).
